# Detection of haemoparasites in selected Australian reptiles using archived blood smears

**DOI:** 10.1007/s00436-026-08683-5

**Published:** 2026-05-13

**Authors:** Rachel Bracken, Diane P Barton, Shokoofeh Shamsi

**Affiliations:** School of Agricultural, Environmental and Veterinary Sciences, Gulbali Institute, Charles Sturt University, Wagga Wagga, NSW Australia

**Keywords:** Australian reptiles, Haemoprotozoa, Leech vectors, Archived blood smears, Retrospective parasitology, Parasite–host associations

## Abstract

Haemoparasites of Australian reptiles remain poorly documented, with much of the available knowledge derived from studies conducted several decades ago. Using archived blood smears, this study examined the occurrence, morphology, and host associations of haemoprotozoans in two gecko species (*Gehyra dubia* and *Hemidactylus frenatus*), two freshwater turtle species (*Emydura macquarii krefftii* and *Muchelys latisternum*), and an associated turtle leech (*Placobdelloides bancrofti*) from northern Queensland, Australia. A total of 332 gecko blood smears were examined, with no blood parasites detected, suggesting that infections were absent, rare, or below the detection threshold at the time of sampling. In contrast, protistan parasites were detected in 93% of 15 freshwater turtles examined, with high prevalence of both haemogregarines and trypanosomes and frequent co-infections. Morphological characteristics and developmental stages of haemogregarines were consistent with the genus *Haemogregarina*, and similar with *Haemogregarina clelandi*, representing a new geographic record. Trypanosome infections occurred as multiple trypomastigote morphotypes consistent with previous descriptions of *Trypanosoma chelodinae* and related forms. All 38 leech crop smears examined were positive for *Trypanosoma* spp., predominantly at the epimastigote stage, supporting the role of *P. bancrofti* as a vector of turtle trypanosomes in Australian freshwater systems. This study highlights marked contrasts in haemoprotozoan occurrence between terrestrial and aquatic reptiles, reinforces the importance of leeches in turtle parasite transmission, and demonstrates the value of archived blood smears for establishing historical baselines and addressing knowledge gaps in Australian reptile parasitology.

**Supplementary Information:**

The online version contains supplementary material available at 10.1007/s00436-026-08683-5.

## Introduction

Australia supports one of the most diverse reptile faunas globally, with approximately 1120 described species (http://www.reptile-database.org/db-info/diversity.html), representing nearly 10% of the world’s reptilian biodiversity (Adlard and O’Donoghue 1998; Cogger 2014). These species occupy a wide range of ecological niches, spanning arid inland deserts, tropical rainforests, freshwater systems, and marine environments (Cogger 2014). Despite this ecological breadth, our knowledge of reptile parasites remains limited and fragmentary, with available information largely restricted to helminths (for example, Nicoll, 1918; Barton et al. 2022). Comparatively more is known about helminth parasites because many are larger, more readily detected during examination, and in some cases associated with visible pathological changes or gross lesions (Barton et al., 2020). In Australia, reptile parasites, particularly blood-dwelling protists, remain poorly documented relative to those of reptiles in other regions (Adlard and O’Donoghue 1998; Telford 2009; Boysen et al. 2022a; Norval et al. 2022; Glassman and Zachariah 2024; Scheelings et al. 2024; Young et al. 2024; Zechmeisterová et al. 2024).

The native gecko *Gehyra dubia* (Macleay, 1877) is widely distributed across eastern Australia and is well adapted to both natural and human-modified environments (Cogger 2014; Bourke et al. 2017). As an insectivore, it consumes a range of invertebrates including mosquitoes, beetles, and spiders (Nordberg et al. 2018), positioning it at the interface between terrestrial food webs and vector populations and susceptible host for parasites. Previous parasitological investigations of Australian geckoes remain limited. Goldberg and Bursey (2021) reported new helminth records from the dubious dtella (*G. dubia*) and the spotted dtella (*Gehyra punctata* (Fry, 1914), expanding the known helminth fauna of these native species. While this study contributed valuable baseline data on gastrointestinal parasites, knowledge regarding the diversity and prevalence of haemoparasites in these hosts remains to be explored.

In contrast, *Hemidactylus frenatus* Duméril & Bibron, 1836, the Asian house gecko, is one of three introduced reptile species established in Australia but has achieved an exceptionally broad distribution globally (West 2018; Valdez-Villavicencio et al. 2024). Since its first Australian record in Darwin in 1960, *H. frenatus* has rapidly expanded across northern Australia and into urban centres along the east coast (Hoskin 2011). This species is known to displace native geckoes and has introduced multiple ectoparasites (*Geckobia* spp.; Barnett et al. 2018) to Australia, highlighting its potential role in parasite transmission and redistribution (West 2018; Valdez-Villavicencio et al. 2024). Despite its invasive success and ecological impact, reports of haemoprotozoan infections in *H. frenatus* remain scarce. However, Telford (2009) notes that *Trypanosoma phlebotomi* was reported from *Hemidactylus frenatus* in Assam, India, with its development studied in the sand fly *Sergentomyia babu shortti* (Shortt and Swaminath 1931). This limited evidence suggests that haemoparasites associated with this species remain poorly documented, particularly in Australia. This absence of data is notable given growing evidence that introduced reptiles can participate in parasite spillover, spillback, or dilution dynamics when they share habitats with native species.

Experimental and field studies across Pacific islands have shown that *H. frenatus* alters the density of native gecko populations and participates in bidirectional parasite exchange, both introducing novel parasites and acquiring native ones (Dame and Petren 2006). In Australia, Barnett et al. (2018) demonstrated that both *G. dubia* and *H. frenatus* can harbour larval stages of the native pentastome belonging to the genus *Waddycephalus* Sambon, 1922, raising concerns about parasite spillback driven by high densities of the introduced host. Although these examples involve non-blood parasites, they highlight the capacity of *H. frenatus* to function as both recipient and amplifier of parasites within Australian ecosystems, underscoring the need to investigate its role in haemoparasite transmission.

Australian freshwater turtles, including *Muchelys latisternum* (Gray, 1867) and *Emydura macquarii krefftii* (Gray, 1871), are widespread along the eastern seaboard and into northern Australia, occupying rivers and streams with permanent water flow (Freeman and Cann 2014). These species exhibit life histories that involve prolonged exposure to aquatic ectoparasites, particularly leeches (McKenna et al. 2005). Leeches of the genus *Placobdelloides* Sawyer, 1986 are known blood-feeding parasites of Australian freshwater turtles and crocodilians (Jakes et al. 2001a; McKenna et al. 2005). *Placobdelloides bancrofti* (Best, 1931) is widely distributed in northern Queensland and the Northern Territory and feeds using a protrusible proboscis characteristic of glossiphoniid leeches. Internationally, leeches are well-established vectors of reptile haemoparasites, including haemogregarines and trypanosomes (Walden et al. 2020), yet their role in parasite transmission within Australian freshwater turtle systems remains poorly resolved.

Recent health investigations of freshwater turtles in north Queensland have documented unexplained cutaneous lesions with strong seasonal and age-related patterns (Wirth et al. 2020). Although leeches were recorded during these studies, their potential role as vectors or contributors to disease processes was not examined in detail. This omission reflects a broader tendency for parasites and their vectors to be under-represented in multidisciplinary wildlife health investigations, despite their known capacity to influence host health and disease emergence.

To date, only 69 protistan parasites, identified to species, have been reported from Australian reptiles, with a large number of unidentified species across all major genera (Adlard and O’Donoghue 1998). It has been estimated that the true diversity of protistan parasites in Australian reptiles may exceed 200 species, indicating a substantial under-representation in the literature. Much of the foundational work on Australian reptile haemoparasites dates back more than half a century, with relatively few contemporary studies providing updated baseline data (Mackerras 1961; Peirce and Adlard 2004).

Since those early investigations, Australian ecosystems have undergone significant change, including the establishment and spread of introduced reptile species, increased urbanisation, and the impacts of climate change and extreme weather events. Establishing baseline data on parasite prevalence is therefore essential for understanding host–parasite dynamics, detecting emerging disease risks, and interpreting future ecological change (Perkins et al. 2009; Boysen et al. 2022b).

Archived blood smears represent an underutilised but valuable resource for retrospective parasitological surveillance. When appropriately prepared and preserved, such smears allow detection of haemoparasites, estimation of parasitaemia, and assessment of host–parasite associations without the need for additional animal sampling. This approach is particularly relevant for protected or logistically challenging wildlife species and enables historical comparisons across time and space.

The present study examines archived blood smears obtained from two gecko species, *G. dubia* and *H. frenatus* (collected for Machado (1998), two freshwater turtle species, *E. m. krefftii* and *M. latisternum*, and the turtle leech *P. bancrofti* (collected for McKenna et al. (2005). The aims were to (i) screen archived smears for the presence of haemoprotozoans and other blood parasites, (ii) document parasite morphotypes and host associations, and (iii) explore the potential role of invertebrate vectors in parasite transmission within these Australian reptile systems.

## Materials and methods

All material examined in this study comprised archived samples collected during previously approved research projects, and no animals were handled or sampled specifically for this study. Original animal ethics approvals were granted by the relevant institutional Animal Ethics Committees at the time of collection (Approval IDs: A693; A755-02), with associated state permits (WISP00283102). Retrospective examination of archived material was approved by the Charles Sturt University Animal Ethics Committee (Approval No. 2024EA-03). At the time of collection, unfortunately, no further samples (host blood and/or liver) were kept for molecular analyses. Following collection, all archived samples were retained in the personal collection of one of the authors (DPB) until examination for this study. Representative samples were deposited in the collection of the Queensland Museum (museum accession number G466306 to G466360). Terminology relating to Protista and protozoa follows the definitions outlined by (O’Donoghue 2017), recognising the historical and functional use of these terms in parasitological literature.

### Geckoes sampling

**Fig. 1:**
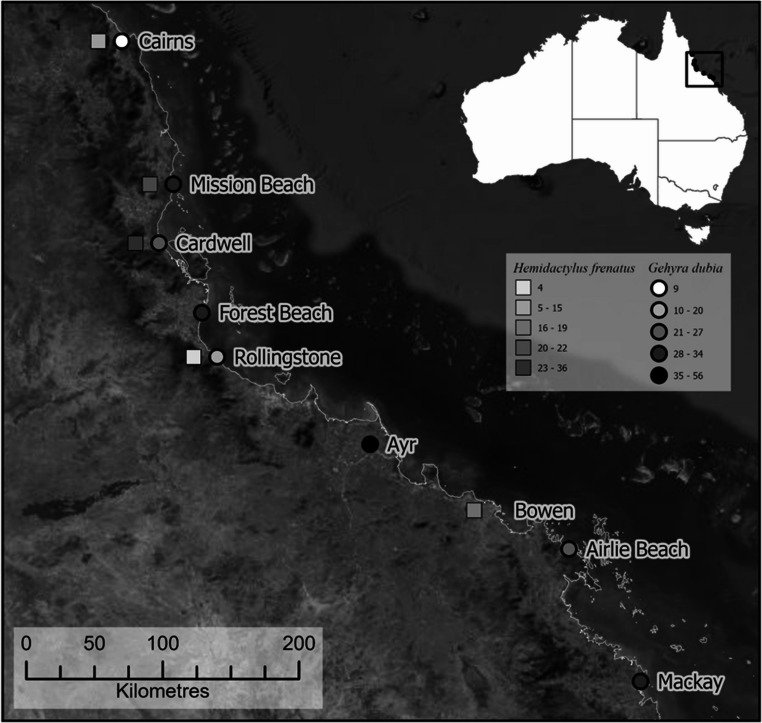
Map of northern Queensland, Australia, showing gecko sample collection locations. (Created by CSU SPAN)

Field collections were carried out between February and May 1998 from ten locations in northern Queensland, Australia, with a minimum distance of 20 km between sampling sites to ensure independent population sampling (Fig. 1) ( Machado (1998). Collection localities included Airlie Beach, Ayr, Bowen, Cairns, Cardwell, Forest Beach, Mackay, Mission Beach, Rollingstone, and Townsville (Table 1).

**Table 1:** Location and number of slide Samples from geckos in the present study

Location		No of collected samples	
	Lat/Long	Gehyra dubia	Hemidactylus frenatus
Airlie Beach	-20.267500 S 148.716949E	27	-
Ayr	-19.576660 S 147.405823E	56	-
Bowen	-20.012440 S 148.246368E	-	19
Cairns	-16.922779 S 145.770294E	9	15
Cardwell	-18.252857 S 146.015035E	25	36
Forest Beach	-18.709901 S 146.298301E	33	-
Mackay	-21.139624 S 149.188175E	32	-
Mission Beach	-17.864886 S 146.109595E	34	22
Rollingstone	-19.002415 S 146.397734E	20	4
Total		236	96

Geckoes were captured from buildings with artificial lighting, placed individually into cloth bags, and transported to the research facility in a cooled esky. Following euthanasia, a small (< 0.5 ml) blood sample was collected from the heart using a sterile insulin syringe and a thin blood smear prepared. A total of 332 Giemsa-stained gecko blood smears were produced (Table 1). Due to the presence of extensive ectoparasite and endoparasite infections, these blood smears were not examined as part of the original study and were archived for future parasitological investigation.

### Turtle and leech sampling

Archived turtle and leech blood smears originated from a research project conducted in 2001 (McKenna et al. 2005). Thirty-one freshwater turtles were captured from the Ross River, Townsville, Queensland (-19.316988 S, 146.751149E), using bait traps and scoop nets. Approximately 0.5 ml of blood was collected from each turtle by puncturing the caudal vein with a sterile insulin syringe prior to their release at the site of capture; thin blood smears were prepared on location. During handling, approximately 400 leeches were removed from turtles and maintained in containers of river water for further examination; at time of processing a subset of leeches were dissected and a series of leech crop smears prepared. The blood and leech crop smears were excluded from the original study due to its focus on the redescription and expanded distribution of the leech species *P. bancrofti* and were archived for subsequent parasitological assessment.

### Microscopic examination of blood smears

All blood smears were examined using a Nikon Eclipse Ci light microscope equipped with a Nikon digital camera and NIS-Elements imaging software (Nikon, Tokyo, Japan). Initial screening was conducted at 400× magnification in areas containing a single layer of blood cells for 20–40 min per smear to assess the presence or absence of haemoparasites. Areas exhibiting suspected parasitic stages or abnormalities were subsequently examined at 1000× magnification under oil immersion to facilitate parasite identification and to distinguish parasites from artefacts (Jakes et al. 2001b; Pineda-Catalan et al. 2013; González et al. 2019).

### Data handling and analysis

Data were collated in Microsoft Excel, with a checklist used to record slide identification, host species, and parasite presence or absence. Notes on smear quality and unusual or unidentified findings were recorded to guide further assessment. Representative photomicrographs of parasitic stages were compiled using Microsoft PowerPoint to facilitate comparative review.

Given the absence of associated host health data and the lack of reported clinical manifestations in the sampled animals, measures of infection intensity and severity were not assessed. Parasite occurrence was summarised as prevalence, expressed as the proportion of positive smears within each host group and presented as fractions and percentages. Species-based and sex-based prevalence values were calculated where applicable (Jakes et al. 2001b). Statistical analyses were not performed due to the limited sample size and variation.

### Morphological identification and measurements

**Fig. 2:**
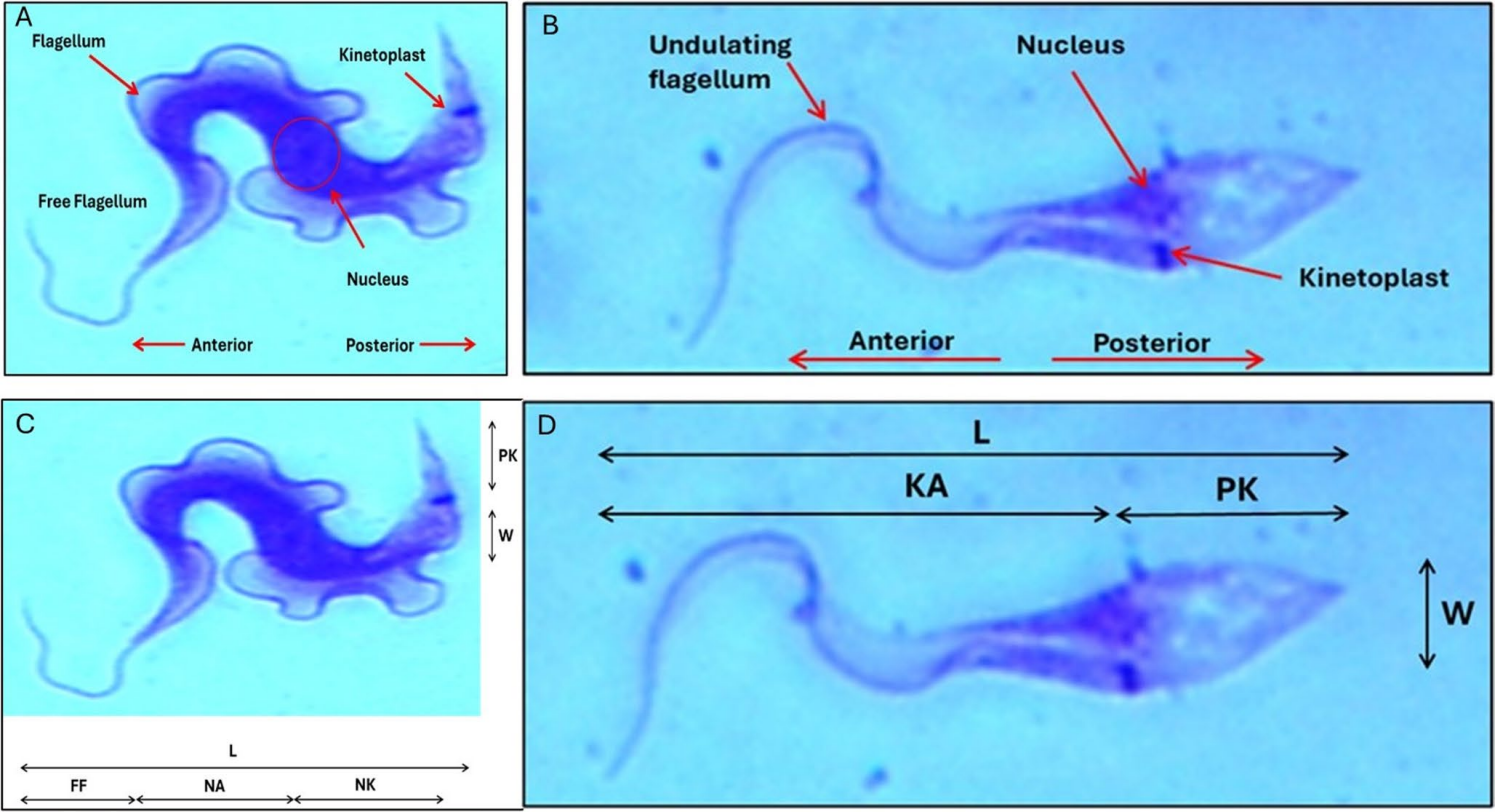
Morphological diagrams and measurement landmarks for *Trypanosoma* spp. (**A**) Trypomastigote morphology; (**B**) trypomastigote measurement points: total length (L), width (W), posterior end to kinetoplast (PK), kinetoplast to nucleus (NK), nucleus to anterior end (NA), and free flagellum length (FF). (**C**) Epimastigote morphology; (**D**) epimastigote measurement points: total length (L), width (W), posterior end to kinetoplast (PK), and kinetoplast to anterior end (KA)

Parasites were identified based on morphological characteristics observable in blood smears (Fig. 2), including the presence of kinetoplasts, parasite developmental stages, and relative size, following established descriptions and taxonomic frameworks (Hoare 1972; O’Donoghue and Adlard 2000; Jakes et al. 2001b; O’Donoghue 2017). Putative sexual stages were interpreted based on morphological criteria described in Kreier (2013), including differentiation of microgamonts and macrogamonts in blood smears. Where definitive genus-level identification was not possible, parasites were assigned to morphotype categories.

Morphometric measurements of trypomastigotes followed the criteria proposed by Hoare (1972) and applied by Jakes et al. (2001b), including total length (L), width (W), distance from nucleus to kinetoplast (NK), kinetoplast to posterior end (PK), distance from anterior end to nucleus (NA), and free flagellum length (FF). Measurements were recorded in micrometres (µm), and arithmetic means were calculated. Anatomical reference diagrams and measurement landmarks are provided in Fig. 2.

## Results

### Hosts and prevalence

#### Geckos 

A total of 332 blood smears from *G. dubia* and *H. frenatus* were systematically examined (Table 1); however, no haemoprotozoans, microfilariae, or other blood-borne parasites were observed in any of the smears.

#### Freshwater turtles

 A total of 25 Giemsa-stained blood smears representing 15 individual freshwater turtles (Table 2) were of sufficient quality to allow examination for the presence of haemoprotozoan parasites. In total, 24 blood smears were examined from 14 *Emydura macquarii krefftii* (11 females and 3 males), and one blood smear was examined from a single *M. latisternum* individual (male).

**Table 2:** Slide examination combined results of parasite detection

Turtle species	Turtle ID	Host Sex	Haemogregarina	Trypanosoma
*Emydura macquarii kreftii*	373	F	-	+
	376	F	-	-
	385	F	-	+
	387	F	-	+
	299	F	+	-
	374	F	+	+
	377	F	+	+
	378	F	+	-
	384	F	+	+
	386	F	+	-
	56	F	+	-
	360	M	+	+
	375	M	+	+
	39	M	+	+
*Muchelys latisternum*	ELM	M	-	+

Haemoprotozoan parasites were detected in 24 (96%) of the 25 blood smears examined, corresponding to infections in 14 (93%) of the 15 individual turtles (Table 2). Haemogregarine infections were identified in 10 (67%) of the turtles, while trypanosome infections were also detected in 10 (67%) of individuals. Co-infections with both haemogregarines and trypanosomes were observed in six turtles (40%). Haemoprotozoan parasites, including *haemogregarine and trypanosome* were detected in 7 (64%) of 11 female turtles and in all 4 male turtles (100%). Among females, haemogregarine infections were detected in all infected individuals, while trypanosome infections were present in six females (55%); three females (27%) exhibited co-infection. All male turtles were infected with trypanosomes; haemogregarine infections were detected only in *E. m. krefftii*, with all three infected males exhibiting co-infection.

#### Morphometric identification of *Haemogregarina*spp. in freshwater turtles

**Fig. 3:**
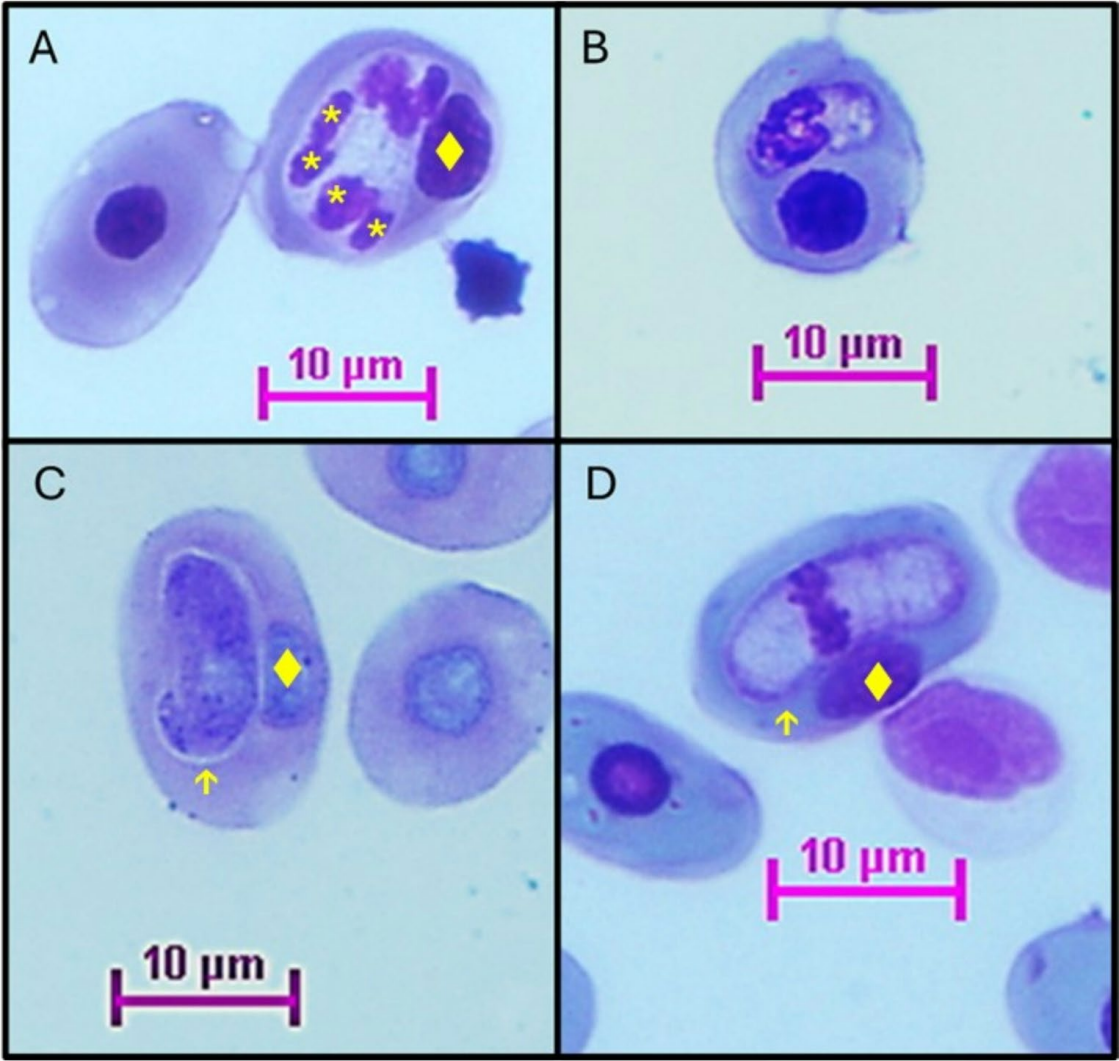
Developmental stages of *Haemogregarina* spp. observed in turtle blood smears. (**A**) Intra-erythrocytic schizont; (**B**) immature gamont; (**C**) male microgamont; (**D**) female macrogamont. Selected merozoites within schizonts are indicated by asterisks. The host erythrocyte nucleus is indicated by a diamond, and parasite nuclei are indicated by arrows

Haemogregarines were identified as belonging to the genus *Haemogregarina* based on the presence of intra-erythrocytic meronts observed in blood smears (Fig. 3A). Meronts were small, elongated ovoid in shape, and contained up to six merozoites. Multiple intra-erythrocytic gamont stages were observed. Immature gamonts were round to ovoid, with a centrally positioned nucleus. Mature gamonts were ovoid to crescent-shaped, with a central to polar nucleus. Microgamonts were characterised by reflexed tails, diffuse nuclei, and paler cytoplasmic staining, whereas macrogamonts exhibited rounded tails, compact nuclei, and darker cytoplasmic staining. Immature gamonts (Fig. 3B) were observed in two individuals whereas mature gamonts (Fig. 3C, D) were detected in 10 turtles. All developmental stages were associated with lateral displacement of the host erythrocyte nucleus. Morphometric measurements of *Haemogregarina* stages are summarised in Table 3.

**Table 3:** Measurements of Haemogregarina stages in micrometres (µm); Note Jakes et al. 2001b did not state the number of measurements

Haemogregarina Stage	Length Mean (Range; Number Measured)		Width Mean (Range)		Nucleus Length Mean (Range)		Nucleus Width Mean (Range)	
	Present study	Jakes et al. 2001b	Present study	Jakes et al. 2001b	Present study	Jakes et al. 2001b	Present study	Jakes et al. 2001b
Mature Gamonts (µm)	12.41 (10.54–14.04; *n* = 19)	12.3 (7.0–15.0)	4.6 (3.05–5.93; *n* = 19)	5.7 (4.0–8.0)	4.44 (3.05–5.36; *n* = 19)	5.3 (3.0–7.0)	2.98 (2.22–4.13; *n* = 19)	4.6 (3.0–6.0)
Immature Gamonts (µm)	8.05 (7.16–10.78; *n* = 13)	9.2 (7.0–12.0)	3.51 (1.82–5.19; *n* = 13)	4.6 (3.0–7.0)	4.06 (2.47–5.41; *n* = 13)	4.5 (2.0–7.0)	3.51 (1.82–4.30; *n* = 13)	4.1 (3.0–6.0)
Schizont Merozoites *	5.86 (4.51–7.03; *n* = 6)	15 (15.2–16.0)	1.71 (1.16–2.30; *n* = 6)	7.1 (7.0-7.2)	-	-	-	-

*Present study measured merozoites inside the shizont, Jakes et al., measured shizont exterior

#### Morphometric identification of *Trypanosoma*trypomastigotes in freshwater turtles

**Fig. 4:**
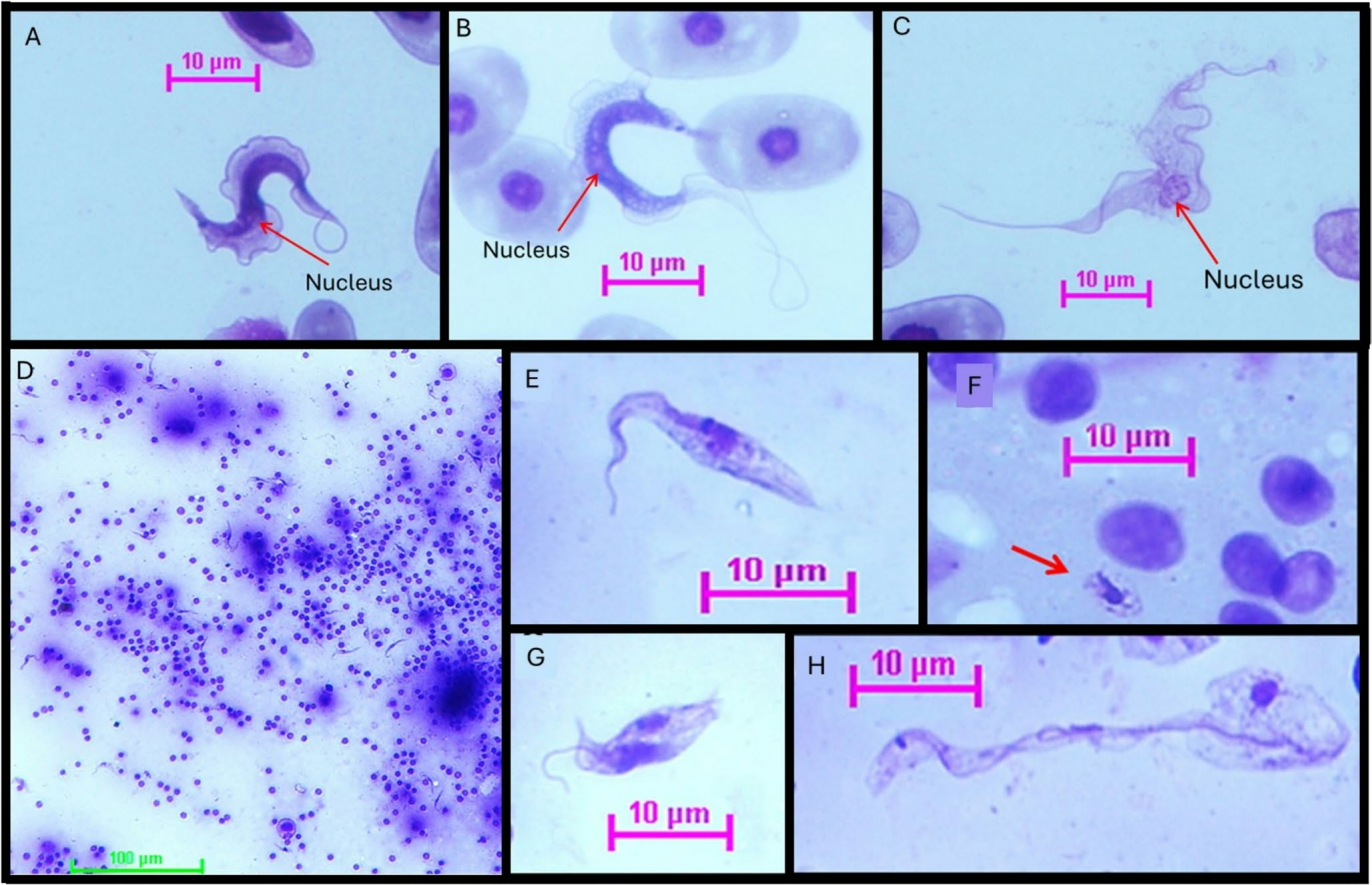
Morphotypes and developmental stages of *Trypanosoma* spp. observed in turtle blood smears and leech crop smears. (**A**) *Trypanosoma* sp. trypomastigote, slender morphotype; (**B**) *Trypanosoma chelodinae* trypomastigote, large morphotype; (**C**) degenerative trypomastigote, degraded morphotype; (**D**) trypomastigotes observed in a leech crop smear at 200× magnification; (**E**–**H**) developmental stages observed in leech crop smears: (**E**) epimastigote, slender morphotype; (**F**) amastigote, round morphotype; (**G**) promastigote, “stingray” morphotype; (**H**) Undetermined, “jellyfish” morphotype

Three distinct trypomastigote morphotypes were recognised (Fig. 4): large, slender, and degraded forms. Large trypomastigotes exhibited greater overall length and increased distance between the kinetoplast and nucleus. Slender trypomastigotes were smaller, with reduced body width and shorter distances between the kinetoplast and nucleus. Degraded trypomastigotes displayed increased body length and greater nucleus–kinetoplast separation compared with both large and slender morphotypes, consistent with morphological distortion or degeneration (Table 4).

**Table 4:** Recorded measurements of trypomastigotes in Micrometres (µm). Note: L (Length), W (Width), PK (Posterior end to Kinetoplast), NK (Kinetoplast to Nucleus), NA (Nucleus to Anterior point), FF (Free Flagellum), measurements given in micrometres (µm); Note Jakes et al. 2001b did not state the number of measurements. NS: not stated

Trypomastigote Description	L Mean (Range; Number Measured)		W Mean (Range; Number Measured)		PK Mean (Range; Number Measured)		NK Mean (Range; Number Measured)			NA Mean (Range; Number Measured)		FF Mean (Range; Number Measured)	
	Present study	Jakes et al. 2001b	Present study	Jakes et al. 2001b	Present study	Jakes et al. 2001b	Present study	Jakes et al. 2001b	Present study		Jakes et al. 2001b	Present study	Jakes et al. 2001b
Large	54.8; (42.8–80.9; *n* = 12)	NS (55–70)	3.3 (2.7–4.5; *n* = 12)	NS (6–7)	5.9 (3.6-8.0; *n* = 12)	NS (3–7)	11.6 (9.1–15.5; *n* = 12)	NS (12–20)	18.5 (12.7–25.1; *n* = 12)		NS (15–23)	18.8 (9.1–36.5; *n* = 12)	NS (17–28)
Slender	47.6 (25.8–60.7; *n* = 5)	NS (52–62)	2.8 (1.2–3.9; *n* = 5)	NS (3–6)	5.4 (3.1–6.8; *n* = 5)	NS (3–6)	7.1 (4.5-9.0; *n* = 5)	NS (3–9)	19.9 (9.9–27.7; *n* = 5)		NS (20–28)	15.2 (8.2–18.8; *n* = 5)	NS (15–24)
Degraded	65.5 (45.4–76; *n* = 3)	NS (52–83)	6.9 (6.1–8.2; *n* = 3)	NS (3–8)	1 (0–3; *n* = 3)	NS (0–7)	24.3 (18–32; *n* = 3)	NS (10–34)	35.5 (27.4–40.9; *n* = 3)		NS (20–28)	12 (8–15; *n* = 3)	NS (14–29)

#### Leeches 

A total of 38 leech crop smears were examined for the presence of haemoprotozoan parasites (Table 5). All smears (100%) were positive for *Trypanosoma* spp. (Fig. 4D to H) Developmental stages observed were predominantly epimastigotes, with promastigote stages also detected.

**Table 5:** Recorded measurements of life stages in leech in micrometres (µm)

Life stages in leech (Description)	Length Mean (Range)	Width Mean (Range)	Kinetoplast to Posterior Mean (Range)	Kinetoplast to Anterior Mean (Range)	Free Flagellum Mean (Range)
‘Jellyfish’ (*n* = 5) (long undulating flagellum, bulbous body)	32.1 (18.9–42.7)	6.3 (5–8)	3.2 (2-4.9)	28.9 (16.3–37.8)	-
Round (*n* = 7) (small, round, no flagellum)	9.4 (5.4–12.7)	6 (5.2–7.8)	-	-	-
Slender (*n* = 27) (thin, long, undulating flagellum)	24.4 (16.2–37.3)	3.8 (1.5–6.9)	7.2 (3–14)	17.2 (8.2–29.6)	-
‘Stingray’ (*n* = 17) (round, large, small flagellum, no undulating membrane)	15.4 (7.7–23.6)	7 (4-9.2)	5.1 (3.8–7.9)	8 (3.3–19)	5.9 (1.5–10.2)

#### *Trypanosoma*stages in leeches

Epimastigotes exhibited a slender body form, with the kinetoplast positioned adjacent to or anterior to the nucleus, and possessed a short free flagellum associated with a small undulating membrane. Promastigote stages were characterised by a more rounded body shape, an anteriorly positioned kinetoplast, a short emergent flagellum, and the absence of an undulating membrane. Seven of the amastigote stage were observed; this stage is typically associated with the gut wall rather than the crop and is therefore less frequently detected in smear preparations. Trypomastigote stages, which are characteristic of vertebrate hosts, were not observed in leech crop smears.

## Discussion

This study provides an assessment of haemoprotozoan parasites in selected Australian reptiles and an associated invertebrate vector based on the examination of archived blood smears. While haemoprotozoan parasites were not detected in the examined geckoes, infection was frequently observed in freshwater turtles and their leech parasites. These observations provide baseline information and highlight potential ecological contrasts in haemoparasite occurrence among reptile host groups, although the absence of molecular confirmation and the variable quality of archived smears may influence detection.

The known haemogregarine parasites of Australian geckos, include *Haemogregarina heteronotae* from *Heteronotia binoei*, commonly known as Bynoe’s gecko, and unknown species of *Haemogregarina* from *Gehyra robusta* (robust dtella), *Gehyra variegata* (tree dtella), *Heteronotia binoei* (Bynoe’s gecko), *Nephrurus wheeleri*,* Oedura tyroni* (southern spotted velvet gecko) and *Phyllurus platurus* (southern leaf-tailed gecko) (O’Donoghue and Adlard 2000).

Other than haemogregarines, *Trypanosoma phylluri* has been found in *Phyllurus platurus* (southern leaf-tailed gecko) and a number of hemosporidians have also been reported in Australian geckos (O’Donoghue and Adlard 2000), including *Billbraya australis* in *Phyllodactylus marmoratus* (marbled gecko); *Haemocystidium* sp. in *Oedura tyroni* (southern spotted velvet gecko); *Haemoproteus gehyrae* in *Gehyra australis* (northern dtella); and *Haemoproteus oedurae* in *Oedura castelnaui* (northern velvet gecko). *Haemoproteus mackerrasi* has been recorded from multiple hosts, including *Gehyra variegata* (tree dtella), *Heteronotia binoei* (Bynoe’s gecko), *Oedura tyroni* (southern spotted velvet gecko), and *Phyllurus platurus* (southern leaf-tailed gecko). In addition, *Haemoproteus underwoodsauri* has been reported from *Underwoodsaurus milii* (thick-tailed gecko), and *Pirhemocyton tarentolae* from both *Gehyra variegata* (tree dtella) and *Phyllurus platurus* (southern leaf-tailed gecko). Given these previous records, the absence of haemoprotozoans in geckoes from the present study is noteworthy, despite the large sample size and broad geographic coverage of northern Queensland. In parasitology, negative findings are not without value; rather, they help refine our understanding of host–parasite systems and generate new hypotheses regarding transmission dynamics, host susceptibility, and vector ecology. An important consideration in interpreting this result is the use of archived blood smears derived from different original studies. The smears examined were prepared at different times and potentially by different investigators, and variation in staining protocols (e.g. Giemsa concentration), smear preparation, and long-term storage conditions may have influenced parasite detectability. Although all smears were examined in the present study by a single observer using consistent criteria, differences in slide age and preparation quality could introduce bias, particularly for low-intensity infections. The age of the archived smears and some fading of the Giemsa staining may have reduced the sensitivity for detecting very low-intensity infections. However, the preservation of key haematological features, including erythrocyte nuclei and cellular morphology, suggests that the absence of haemoparasites likely reflects a true lack of detectable infection at the time of sampling rather than methodological failure. While these factors may have reduced sensitivity for detecting very low parasitaemias, they are unlikely to fully account for the complete absence of haemoprotozoans in gecko samples compared to the relatively high prevalence observed in freshwater turtles. These findings indicate that haemoprotozoan infections in these gecko species were either absent, rare, seasonal, or below the detection threshold of smear-based microscopy.

The absence of haemoprotozoans in *H. frenatus* is particularly noteworthy given its invasive success, high population densities, and demonstrated role in parasite spillback involving other parasite taxa (Hoskin 2011; Barnett et al. 2018). While *H. frenatus* has been shown to acquire and transmit native helminths and pentastomes (Barnett et al. 2018), the present results suggest that this capacity may not extend to haemoprotozoans, potentially reflecting limited exposure to competent vectors or intrinsic resistance to blood parasites. Barnett et al. (2018) reported that *H. frenatus* was only infected with introduced mites, which have not been reported to transfer to native geckoes, and that native mites were not reported from the introduced gecko. Molecular screening of contemporary samples, combined with targeted vector studies, would be required to determine whether haemoprotozoan infections occur sporadically or remain genuinely rare in Australian geckoes.

In contrast to the absence of haemoprotozoans in geckoes, freshwater turtles exhibited a markedly different pattern, with frequent detection of haemoparasites across examined samples. Among freshwater turtles, haemogregarines have been widely reported, with *Haemogregarina clelandi* recorded from multiple host species, including *Chelodina expansa* (broad-shelled river turtle), *Chelodina longicollis* (long-necked tortoise), *Chelodina oblonga* (oblong turtle), *Elseya dentata* (northern snapping turtle), *Muchelys latisternum* (saw-shelled turtle), and *Emydura krefftii* (Krefft’s river turtle) and *Emydura macquarii* (Murray turtle). In addition, unidentified *Haemogregarina* species have also been reported from *Chelodina expansa* (broad-shelled river turtle) (O’Donoghue and Adlard 2000). In regards *Haemogregarina clelandi*, it has been originally reported by Mackerras (1961) from both *Muchelys latisternum* and *Emydura macquarii krefftii*, although locality data were limited to “eastern Australia.” Subsequent work by Jakes et al. (2001b) provided morphometric support for the identification of *H. clelandi* in Australian freshwater turtles. The haemogregarines detected in the present study exhibited comparable morphological features, including intra-erythrocytic schizonts and immature and mature gamonts, supporting their putative identification as *H. clelandi*. Morphometric comparisons between the present study and Jakes et al. (2001b) show a high degree of overall concordance for haemogregarine stages, supporting the identification of these parasites as *Haemogregarina clelandi*. Mean length of mature gamonts was nearly identical between studies (12.41 μm vs. 12.3 μm), although specimens in the present study tended to be narrower, with smaller nuclear dimensions. Similarly, immature gamonts in the present study were slightly smaller in both length and width compared to those reported by Jakes et al. (2001b), but remained within comparable ranges. Greater discrepancies were observed for schizont-associated stages, likely reflecting differences in measurement approach, as the present study quantified individual merozoites within schizonts, whereas Jakes et al. (2001b) reported measurements of the entire schizont. Additionally, Jakes et al. (2001b) did not specify sample sizes, which may further limit direct comparison. Overall, despite minor variation in dimensions, the strong overlap in morphometric ranges and agreement in key morphological features supports the taxonomic consistency of these haemogregarines across studies. The detection of these infections in *E. m. krefftii* from the Ross River therefore potentially represents a new geographic record and extends the known distribution of this haemogregarine.

**Fig. 5:**
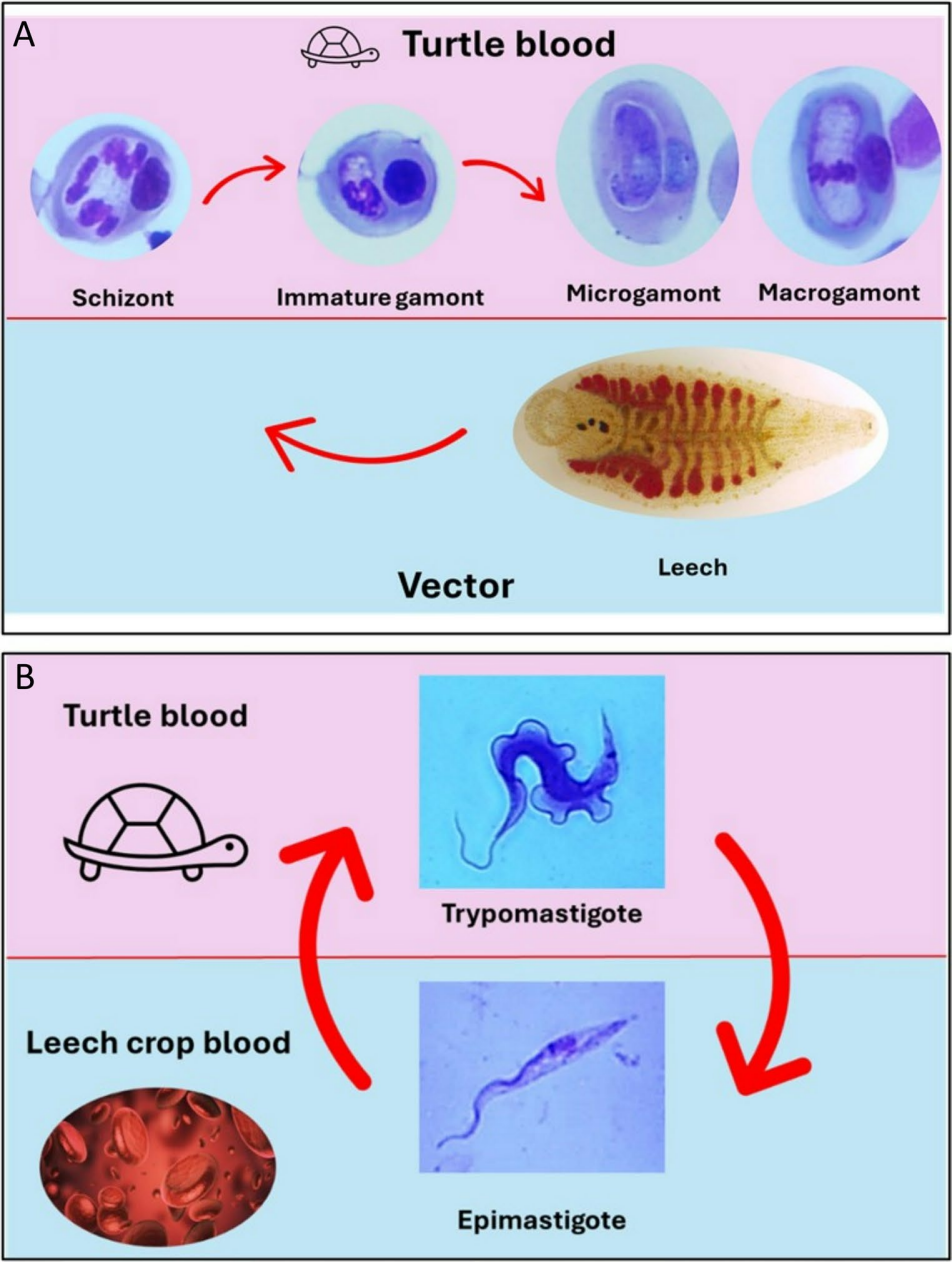
Inferred life-cycle stages of (**A**) *Haemogregarina* sp. and (**B**) *Trypanosoma* sp. based on observations from the present study

While leeches are considered the most likely vectors of *H. clelandi* (Fig. 5A), other haematophagous invertebrates, including biting flies and mosquitoes, may also contribute to transmission, particularly given the mixed basking behaviours of freshwater turtles (Nordberg and McKnight 2020; McKnight et al. 2021). Clarifying vector specificity will require integrated parasitological and entomological investigations.

Trypanosomatid parasites have also been documented in Australian freshwater turtles, with *Trypanosoma chelodina* reported from multiple host species, including *Chelodina longicollis* (long-necked tortoise), *Elseya dentata* (northern snapping turtle), *Muchelys latisternum* (saw-shelled turtle), and *Emydura krefftii* (Krefft’s river turtle) and *Emydura macquarii* (Murray turtle) (O’Donoghue and Adlard 2000). In the present study, trypanosome infections were common in freshwater turtles and occurred as three distinct trypomastigote morphotypes: slender, large, and degraded forms. These morphotypes corresponded closely to those described by Jakes et al. (2001b), with the slender form aligning with *Trypanosoma* sp. (Type 2), the large form with *Trypanosoma chelodinae* (Type 1), and the degraded form with degenerative trypomastigotes (Type 3). Morphometric comparison of trypomastigotes between the present study and Jakes et al. (2001b) (Table 4) reveals general agreement across the three recognised morphotypes (large, slender, and degraded), despite some variation in specific measurements. The large trypomastigotes showed comparable overall lengths between studies, with substantial overlap in ranges, although specimens in the present study tended to be narrower than those reported by Jakes et al. (2001b). Similarly, slender forms in the present study were slightly shorter and narrower on average but remained within the range of values previously described. Degraded forms exhibited the greatest variability, particularly in body width and internal measurements, which is consistent with their degenerative nature and reduced structural integrity. Minor differences in morphometric parameters, including distances between key structures (PK, NK, NA) and free flagellum length, likely reflect a combination of biological variability, differences in parasite condition, and methodological factors such as measurement approach and slide preparation. As with haemogregarines, the absence of reported sample sizes in Jakes et al. (2001b) limits the precision of direct comparison. Overall, the strong overlap in morphometric ranges and consistency in general morphology supports the classification of these trypomastigotes into corresponding morphotypes, while reinforcing the limitations of morphology alone for definitive species-level identification.

The presence of multiple morphotypes likely reflects developmental variation, host immune interactions, or parasite degeneration rather than distinct species. The detection of these morphotypes within the same host population underscores the limitations of morphology alone for species-level identification of trypanosomes and highlights the need for molecular characterisation to resolve taxonomic uncertainty and evolutionary relationships (Elliott et al. 2009; O’Donoghue 2017).

All leech crop smears examined were positive for *Trypanosoma* spp., predominantly at the epimastigote stage, although several promastigote stages were also observed. The high abundance of trypanosome stages within leech crops suggests that conditions within the leech gut are highly conducive to parasite survival and multiplication (Smit et al. 2020). Whether this reflects peak transmission timing or generally favourable microenvironmental conditions remains unclear.

The observation of multiple developmental stages within leeches, coupled with the presence of trypomastigotes in turtle blood, provides strong support for *Placobdelloides bancrofti* acting as a vector of turtle trypanosomes in Australian freshwater systems (Fig. 5B). Although vector competence was not experimentally confirmed, the developmental patterns observed are consistent with established trypanosome life cycles and international studies implicating leeches as definitive hosts (Fermino et al. 2015).

This study demonstrates the considerable value of archived blood smears for parasitological investigations. Such material enables reconstruction of historical parasite distributions and provides baseline data against which future ecological or environmental changes can be assessed. This approach is particularly valuable for wildlife species that are protected, difficult to sample, or under-represented in contemporary surveys (Nakahama 2021; Begum et al. 2024).

However, the opportunistic nature of archived material imposes inherent limitations. Sample sizes, host metadata, and geographic coverage were constrained by original study designs, and some smears were unsuitable for examination due to age-related degradation or preparation techniques. In addition, molecular analyses could not be conducted within the scope of this study, and species-level identifications therefore remain tentative. Future work combining microscopy with molecular tools will be essential to refine parasite taxonomy and explore phylogenetic relationships (Jakes et al. 2001a, b).

Taken together, these findings reveal marked contrasts in haemoprotozoan occurrence between terrestrial geckoes and freshwater turtles in Australia, reinforce the central role of leeches as vectors of turtle trypanosomes, and expand the known geographic distribution of haemoprotozoan infections in Australian freshwater turtles. The results highlight substantial knowledge gaps in Australian reptile haemoparasites and emphasise the need for renewed research efforts using modern diagnostic and molecular approaches.

## Supplementary Information

Below is the link to the electronic supplementary material.

Supplementary Material 1

## Data Availability

No datasets were generated or analysed during the current study.
