# Supplementary material for: Detection of haemoparasites in selected Australian reptiles using archived blood smears
Source: Parasitol Res. 2026 May 13;125(1):78. doi: 10.1007/s00436-026-08683-5 (PMC13341705; doi:10.1007/s00436-026-08683-5)
Supplement: Supplementary file 1 — Supplementary Material 1 [file 436_2026_8683_MOESM1_ESM.docx]

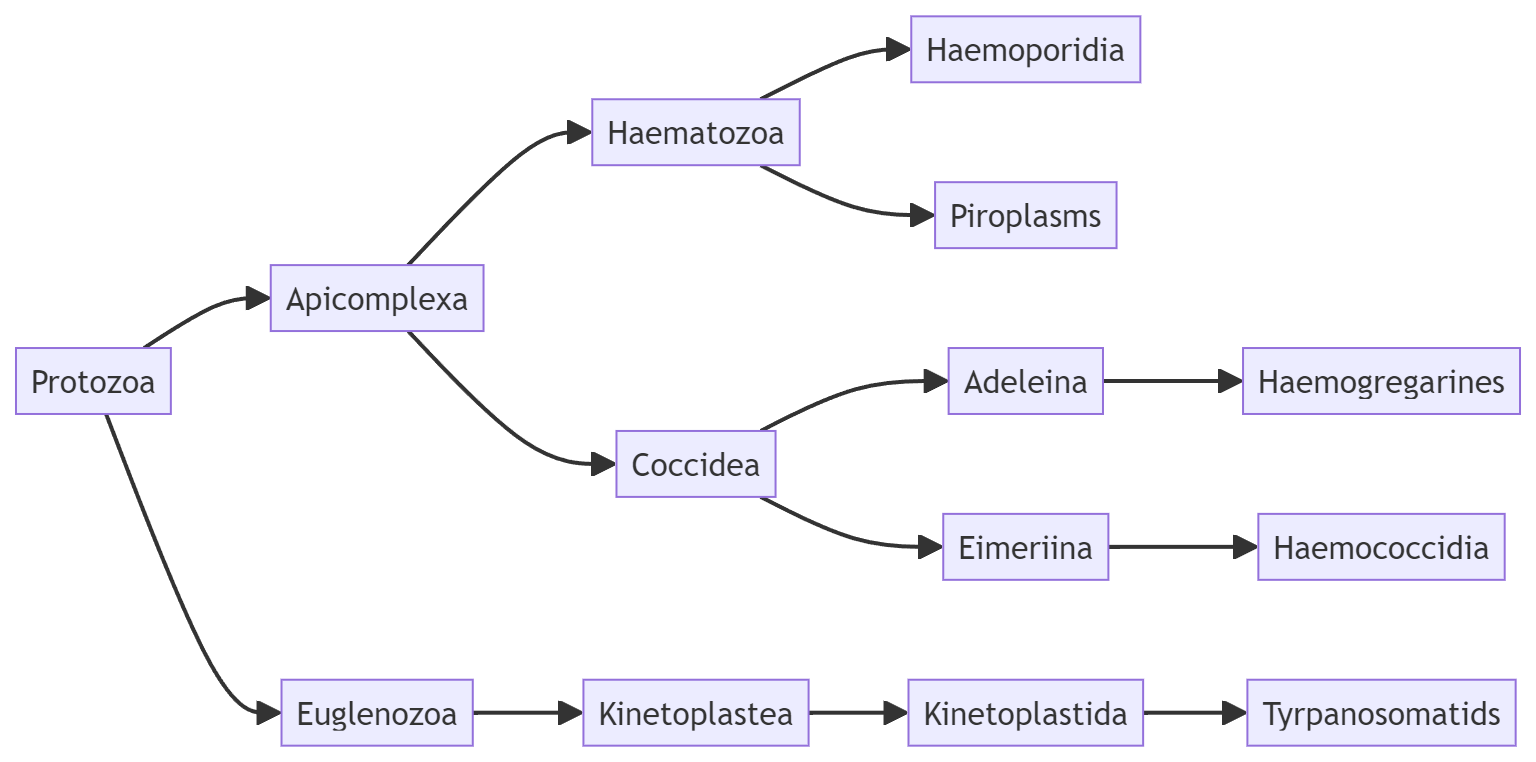


**Figure 1-** Classification assembly of the five Haemoprotozoan parasite categories. Adapted from “Haemoprotozoa: making biological sense of molecular phylogenies”, by (O'Donoghue, 2017), *International Journal for Parasitology, Parasites and Wildlife*, *6*(3), 241-256, (<https://doi.org/10.1016/j.ijppaw.2017.08.007>).


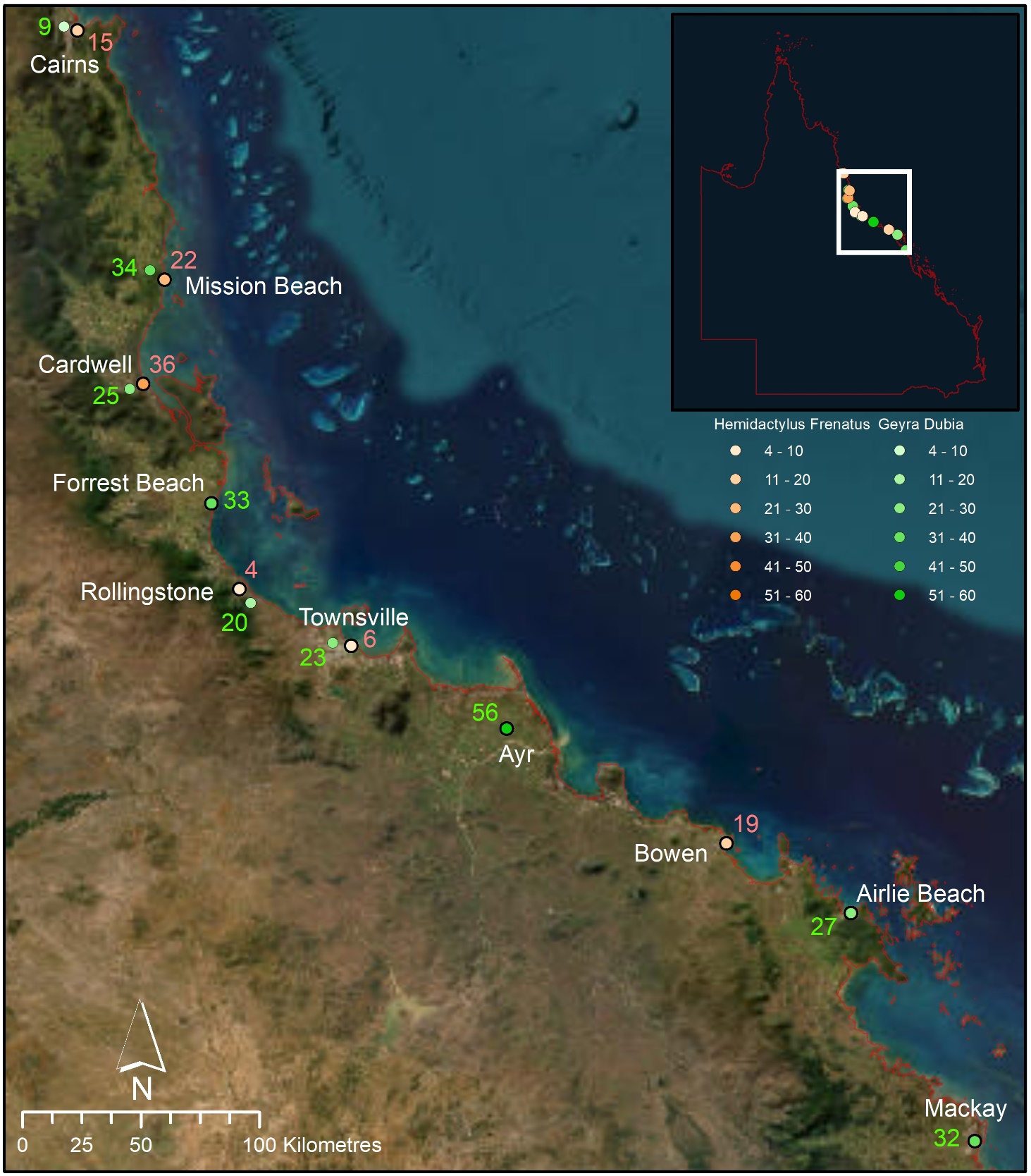


**Figure 2 -** Map of northern Queensland, Australia, showing gecko sample collection locations. *Note*: Green = *Gehyra dubia*, Red = *Hemidactylus frenatus* (Created by CSU SPAN).


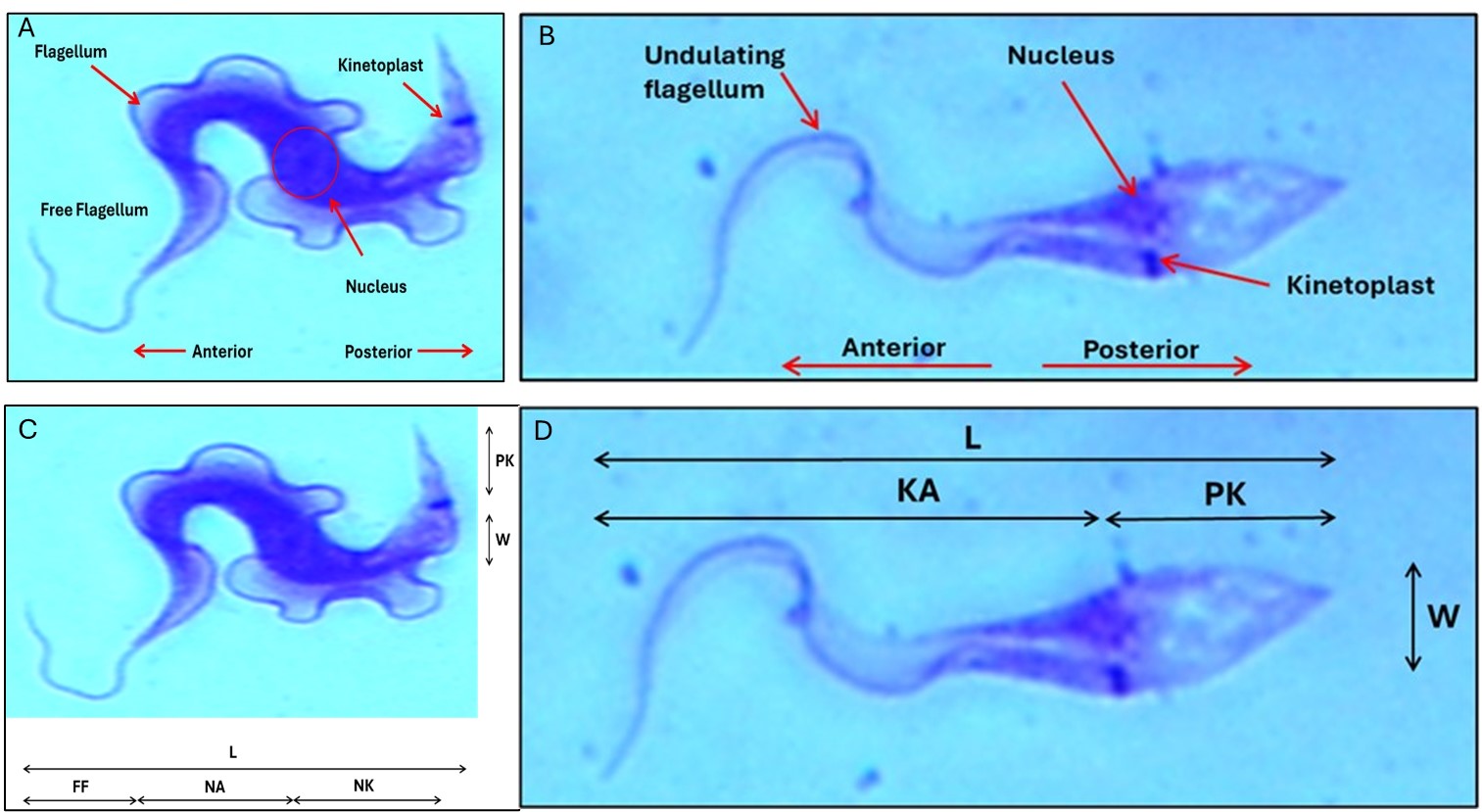


**Figure 3.** Morphological diagrams and measurement landmarks for *Trypanosoma* spp.
(A) Trypomastigote morphology; (B) trypomastigote measurement points: total length (L), width (W), posterior end to kinetoplast (PK), kinetoplast to nucleus (NK), nucleus to anterior end (NA), and free flagellum length (FF). (C) Epimastigote morphology; (D) epimastigote measurement points: total length (L), width (W), posterior end to kinetoplast (PK), and kinetoplast to anterior end (KA).


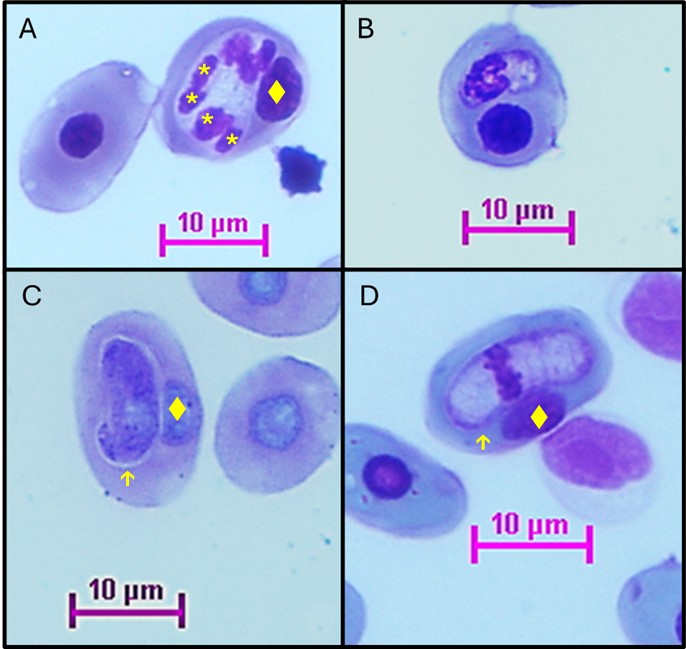


**Figure 4.** Developmental stages of *Haemogregarina* spp. observed in turtle blood smears. (A) Intra-erythrocytic schizont; (B) immature gamont; (C) male microgamont; (D) female macrogamont. Selected merozoites within schizonts are indicated by asterisks. The host erythrocyte nucleus is indicated by a diamond, and parasite nuclei are indicated by arrows.


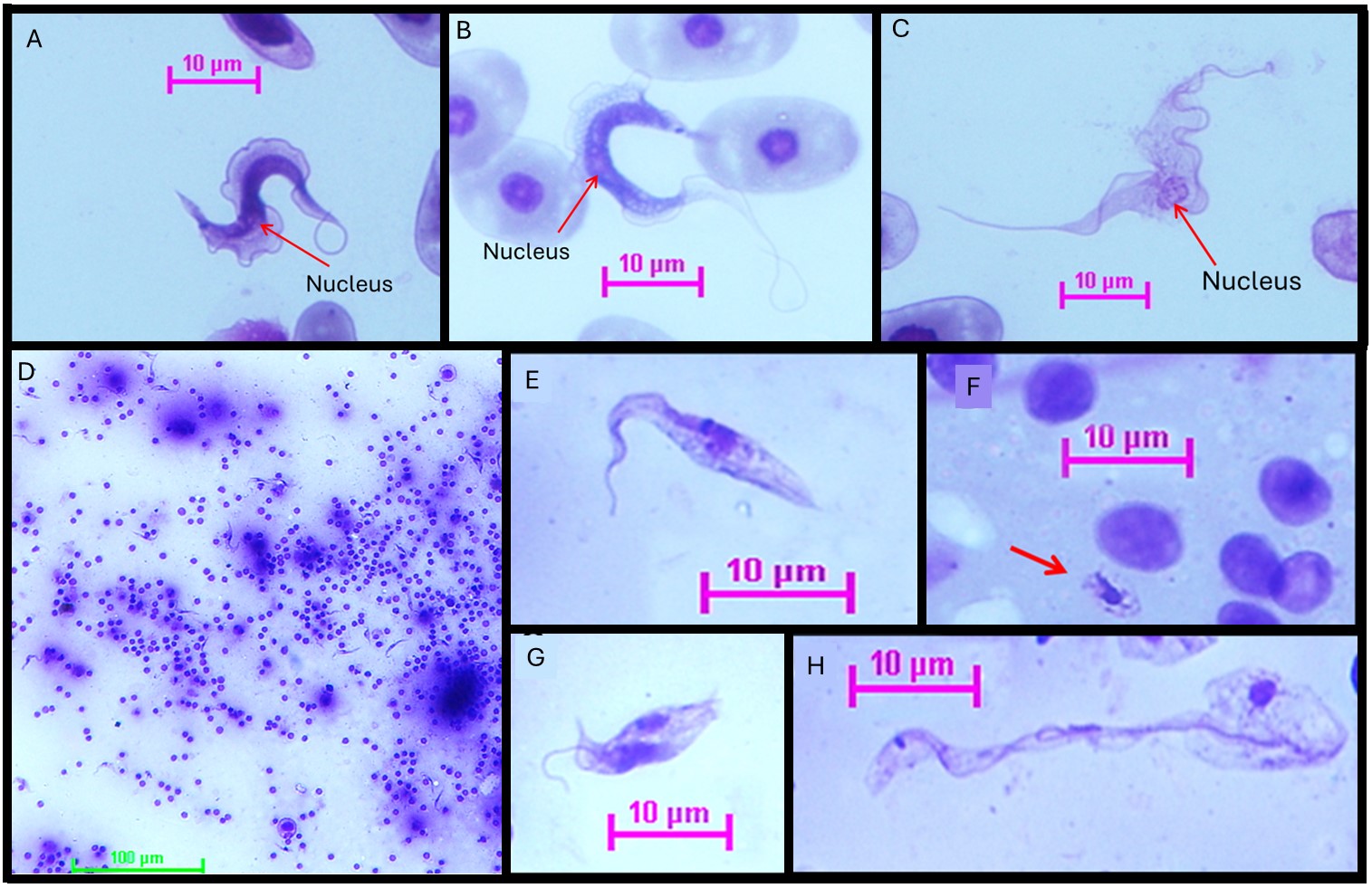


**Figure 5.** Morphotypes and developmental stages of *Trypanosoma* spp. observed in turtle blood smears and leech crop smears. (A) *Trypanosoma* sp. trypomastigote, slender morphotype; (B) *Trypanosoma chelodinae* trypomastigote, large morphotype; (C) degenerative trypomastigote, degraded morphotype; (D) trypomastigotes observed in a leech crop smear at 200× magnification; (E–H) developmental stages observed in leech crop smears: (E) epimastigote, slender morphotype; (F) amastigote, round morphotype; (G) promastigote, “stingray” morphotype; (H) trypomastigote, “jellyfish” morphotype.


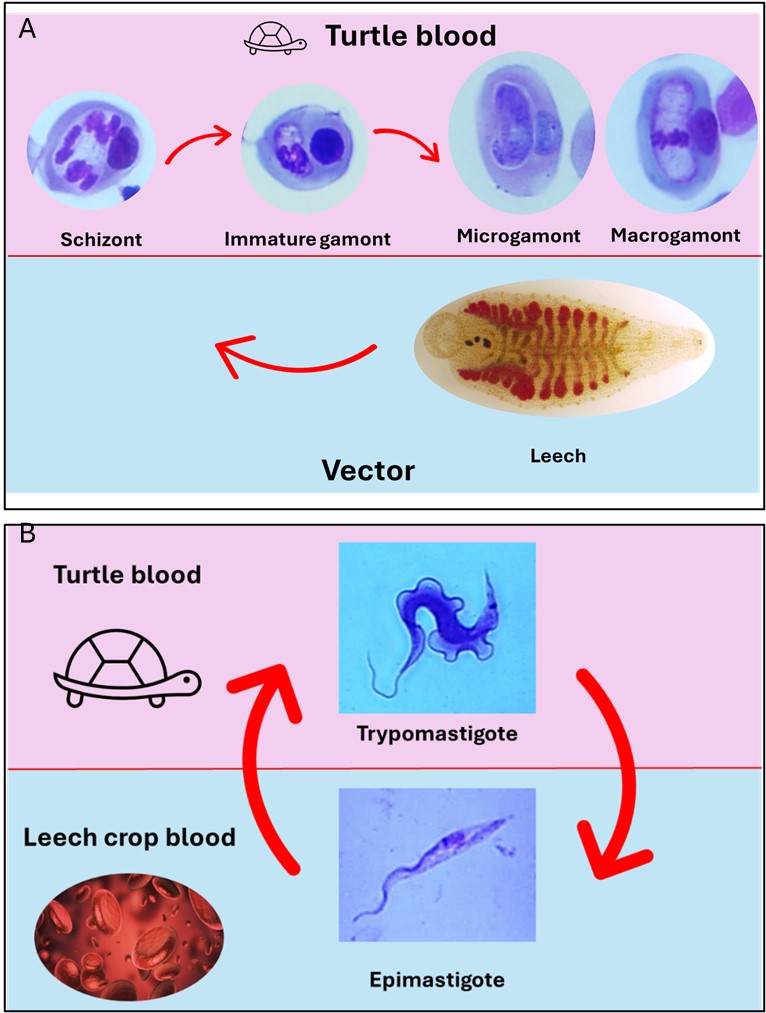


**Figure 5.** Inferred life-cycle stages of (A) *Haemogregarina* sp. and (B) *Trypanosoma* sp. based on observations from the present study.
